# Imaging of clinically silent rectoprostatic hematoma in MRI guided in-bore prostate biopsy

**DOI:** 10.1038/s41598-022-05909-1

**Published:** 2022-02-03

**Authors:** Marietta Garmer, Christin Hoffmann, Dietrich Grönemeyer, Birgit Wagener, Lars Kamper, Patrick Haage

**Affiliations:** 1 Medical Center—Radiology, Bochum, Universitätsstr. 110e, 44799 Bochum, Germany; 2 Witten/Herdecke University, Witten, Germany; 3 Groenemeyer Institute for Microtherapy, Bochum, Germany; 4 Medical Center—Urology, Bochum, Germany; 5 Radiology, Helios University Hospital Wuppertal, Witten/Herdecke University, Witten, Germany

**Keywords:** Prostate, Outcomes research

## Abstract

MR imaging provides awareness for rectoprostatic hematomas as a complication in prostate biopsy. We evaluated the frequency and size of clinically silent bleeding after in-bore MRI-guided prostate biopsy according to documentation in MRI. From 2007 until 2020 in-bore MRI-guided prostate biopsy was performed in 283 consecutive patients with suspected prostate cancer. Interventional image documentation was reviewed for rectoprostatic hematomas and rectal blood collections in this retrospective observational single-center study. Correlation to patient characteristics was analyzed using a multivariable logistic regression model. 283 consecutive patients with a mean age of 66 ± 8 years were included. We diagnosed bleeding complications in 41 (14.5%) of the patients. Significant rectoprostatic hematomas were found in 24 patients. Intra-rectal blood collections were observed in 16 patients and one patient showed bleeding in the urinary bladder. The volume of rectoprostatic hematomas was determined with a median of 7.5 ml (range 2–40 ml, IQR 11.25). We found no correlation between the presence of a rectoprostatic hematoma and malignant findings, patient position in biopsy, number of cores, age, prostate volume nor PSA density (p > 0.05). Rectoprostatic hematomas and rectal blood collections are rare complications after in-bore MR-guided prostate biopsy. MR imaging provides benefits not only for lesion detection in prostate biopsy but also for the control of bleeding complications, which can be overlooked in standard TRUS biopsy. Their significance in pain, erectile dysfunction, and urinary retention remains to be investigated.

## Introduction

Prostate biopsy has recently undergone rapid developments regarding technique and image guidance. For years, the diagnosis of prostate cancer was based on ultrasound-guided systematic biopsies. The accurate lesion detection in ultrasound is limited in the prostate^1^. Magnetic resonance (MR) imaging with delineation of significant lesions is of increasing importance in the diagnostic work-up^2^. The clinical practice guidelines published by the European Society for Medical Oncology and the guidelines on prostate cancer published by the European Association of Urology recommend imaging before prostate biopsy and targeted biopsy in addition to systematic biopsy^3,4^.

Nevertheless, the role of systematic biopsy and the best image-fusion technique is currently discussed^5,6^. The prerequisite for an optimal prostate biopsy strategy is high sensitivity in the detection of significant cancerous lesions combined with a low complication rate. MR-guided prostate biopsy provides high target accuracy and a high detection rate for clinically significant cancer^2,6–8^.

There are different approaches for MR guidance. The easiest way is standard transrectal ultrasound-guided (TRUS) biopsy with a cognitive fusion of MR information. Fusion biopsy uses software for image fusion of MR images to the ultrasound device. True in-bore MR-guided biopsy allows direct image control inside the scanner and is performed via a transrectal, transgluteal, or transperineal approach^2,3,6^. MRI in-bore biopsy might provide a higher per-core percentage of malignant cells compared to TRUS-MRI fusion targeted biopsy with an impact on risk stratification and patient management^9^.

Safety assessments include the rate of complications and the possible need for sedation or general anesthesia. These assessments are complex due to different approaches and different numbers of cores obtained^8^. Nevertheless, safety considerations should also be a key issue, as well as cancer detection in discussions on the best biopsy strategy. Transperineal mapping biopsy leads to a high rate of urinary retention rate and erectile dysfunction but shows a lower rate of infection compared to transrectal biopsy^2,7,10^. In comparison to systematic TRUS biopsy, transrectal in-bore MR-guided targeted biopsy implies a considerably reduced number of cores obtained, and the rate of complications is reported to be lower^6,8,11^.

Bleeding complications can be diagnosed clinically as rectal bleeding, hematuria, or hematospermia. Perineal hematomas are well recognized in transperineal prostate biopsies, they are clinically visible. In contrast, periprostatic hematomas are only recognized by imaging and may be missed as a possible cause of pain or erectile dysfunction^7,12–14^.

The repetitive needle position documentation in transrectal in-bore MR-guided biopsy allows evaluation of collateral periprostatic hematoma; to our knowledge, this however has not yet been evaluated specifically. To date, reviews on complications in prostate biopsy do not include data on periprostatic hematoma^8,15,16^.

Herein we analyzed the imaging for MR guided in-bore prostate biopsy to raise awareness for rectoprostatic hematomas as a complication in prostate biopsy with an initial glance at possible risk factors.

## Methods

Institutional review board approval was obtained by the ethics committee of the Witten/Herdecke University, Germany for this retrospective study. Informed consent was obtained from all patients.

### Patient demographics and clinical data

Multiparametric MRI followed by in-bore MRI-guided biopsy was performed in 283 consecutive patients from 2007 to 2020.

Prostate carcinoma was detected in 173 patients, 110 patients did not show malignancy.

**Figure 1:**
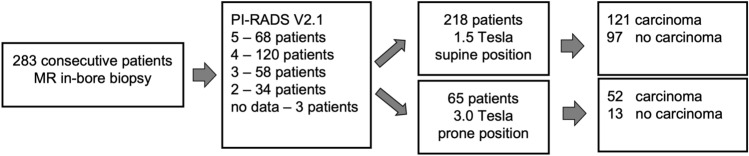
Study workflow.

The retrospectively standardized PI-RADS(V2.1) score according to version 2.1 was 5 in 68 patients, 4 in 120 patients, 3 in 58 patients, and 2 in 34 patients; three patients had missing diagnostic data for retrospective PI-RADS evaluation (Fig. 1).

The mean prostate volume in our patients measured in MRI was 49.7 ml (range 10–175 ml), mean prostate-specific antigen PSA was 9.63 ng/ml (range 0.4–122.7 ng/ml), mean PSA density was 0.22 ng/ccm (range 0.02–2.32 ng/ccm).

### In-bore MR-guided biopsy

MR imaging was performed using a 1.5 Tesla (ESPREE, Siemens Healthcare) or a 3 Tesla (PIONEER, GE Healthcare) scanner with a standard protocol fulfilling the requirements of the Consensus Meeting on the Standardization of Prostate MRI and the appropriate version of the prostate imaging reporting and data system, respectively^1,17^.

Patients were scheduled for biopsy in a consensus-based decision by radiologist and urologist. The concept of targeted in-bore MRI guided biopsy serves as a complement to the standard procedure using systematic biopsies. In contrast to fusions biopsies, in-bore MRI guided biopsy cannot combine targeted biopsy to systematic biopsy. All methods were carried out in accordance with relevant guidelines. Therefore, all patients were offered a systematic biopsy. The decision for a systematic biopsy before or after the targeted biopsy was made on an individual basis. The systematic biopsy was not subject to this study.

Lesions scored PIRADS category 3 or above were assigned to biopsy insofar as a PI-RADS version was available at the time of biopsy. All lesions were reviewed retrospectively according to PI-RADS version 2.1 by an experienced radiologist who performed all biopsies since 2008.

The biopsy was carried out in a second session to allow accurate planning. In the case of oral anticoagulation therapy, a treatment interruption with or without bridging was handled individually. In general, antiplatelet therapy was continued. At least one lesion with the highest suspicion was chosen on the left and the right side of the prostate. In the case of unilateral lesions according to the MRI, a contralateral target area was plotted in the peripheral zone. The position of the targets was documented according to the prostate sectors given in the PI-RADS(V2.1) scheme.

### Image acquisition protocol and analysis

For the biopsy, the patient was positioned using a biopsy positioning device in the supine position in the 1.5 Tesla scanner (preproduction model by Invivo) or the prone position in the 3 Tesla scanner (DynaTRIM by Philips). Intrarectal lidocaine jelly was used for local anesthesia. An adjustable needle guide was inserted transrectally under control of sagittal and coronal fast imaging. Based on an iterative process the alignment of the needle guide was modified according to the biopsy planning. In the correct position, an 18 G MR compatible fully automatic biopsy gun was inserted to obtain the cores. Each biopsy procedure was documented by fast T2-weighted imaging in axial plane, and in sagittal or coronal plane with the needle inside the lesion (Table 1). Where the targeted lesion was not hit sufficiently, biopsies were repeated, so 1–7 cores (median 4) were taken per patient. The prostate sector for each target was documented according to the PI-RADS V2.1 scheme. The needle path was assigned to a lateral path for targets in the basal, the middle, and the apical posterolateral peripheral zone (PZpl). All biopsy specimens were evaluated in the same histopathologic institute including Gleason grading of prostatic carcinoma and immunohistochemistry if applicable.

**Table 1:** Sequence protocol of MRI guided in-bore biopsy.

Field strength	Sequence for needle control	Plane	TR/TE/FA	Slice thickness	Matrix	FOV
1.5 Tesla	TrueFISP for navigation needle holder	Coronal sagittal	4.2 /2.1/57	3.5	256 × 256	400 × 400
	TrueFISP for needle control	Axial	4.5/2.3/57	3.5	384 × 384	400 × 400
	BLADE for needle control	Sagittal	4260/137/148	3.0	320 × 320	420 × 420
3.0 Tesla	FIESTA for navigation needle holder	Coronal sagittal	5.5/2.5/65	3.0	224 × 260	260 × 260
	T2w TSE for needle control	Axial	3500/149/111	3.0	320 × 320	200 × 200
	T2 TSE for needle control	Sagittal	3500/150/130	3.0	256 × 256	200 × 200

*TrueFISP* true fast imaging with steady state precession, *BLADE motion insensitive* multi-shot turbo spin echo sequence, *FIESTA* fast imaging employing steady state acquisition, *TSE* turbo spin echo.

### Measurement of hematomas

**Figure 2:**
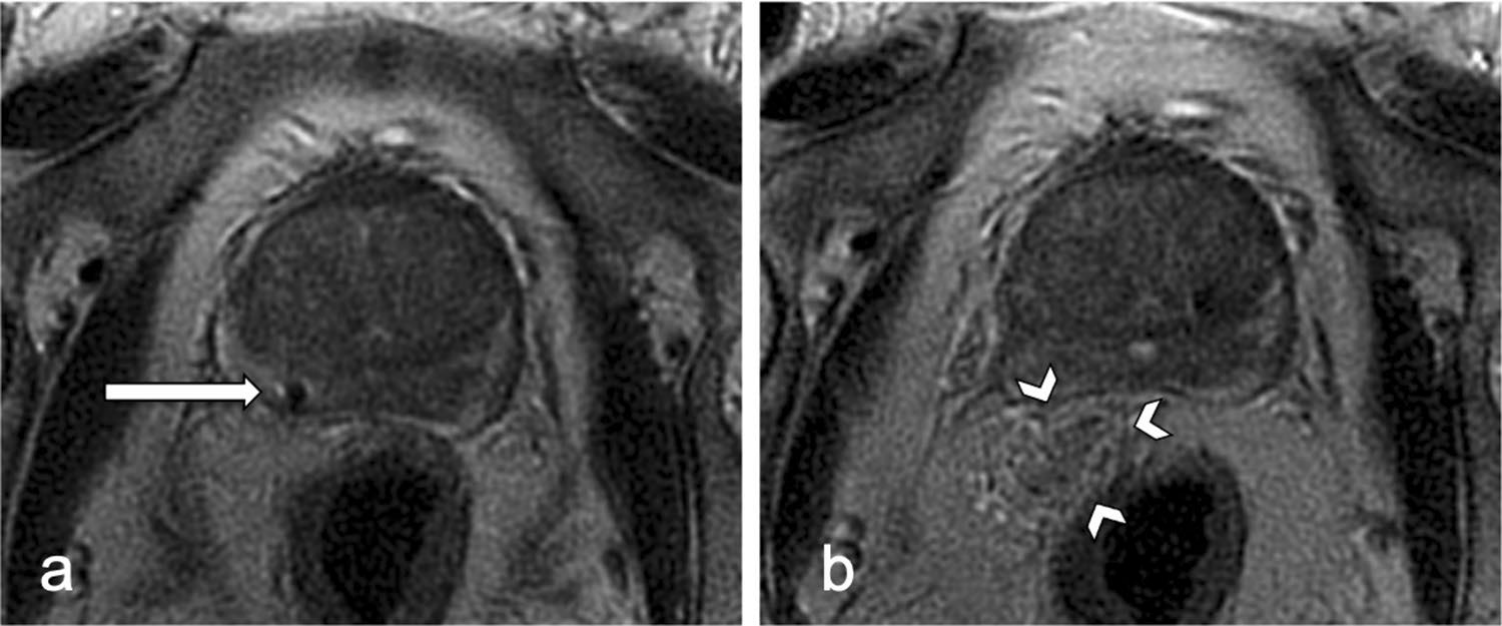
Small rectoprostatic hematoma of 3 ml (arrowheads); axial T2 weighted fast imaging in a 77 years old patient with continued antiplatelet therapy; (**a**) first intraprostatic needle documentation, inside the peripheral zone on the right (arrow); (**b**) needle documentation at the end of the intervention.

**Figure 3:**
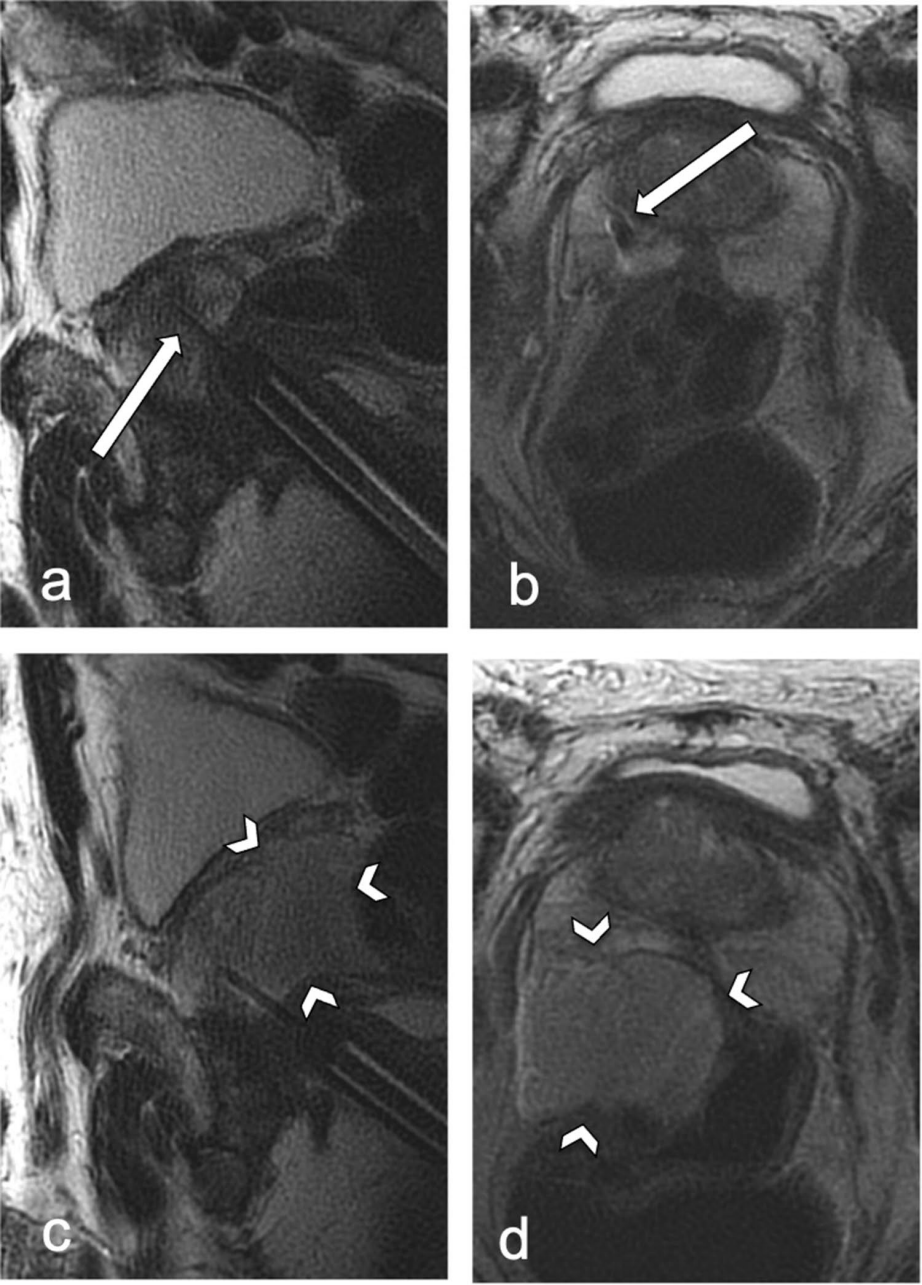
Large rectoprostatic hematoma of 40 ml (arrowheads); sagittal (**a**,**c**) and axial (**b**,**d**) T2-weighted fast imaging in a 63 years old patient without anticoagulants nor antiplatelet therapy; (**a**,**b**) first intraprostatic needle documentation, inside the peripheral zone on the right (arrows); (**c**,**d**) needle documentation at the end of the intervention.

For image analysis, the MR scans were reviewed retrospectively by the same radiologist who performed the biopsies. The last documentation of the needle position at the end of the biopsy procedure was selected and rated for the presence of a hematoma (Figs. 2, 3.). Where appropriate, images were compared to prebiopsy images from the beginning of the procedure. Signs of a hematoma were newly appearing, mostly circumscribed formations with a more or less space-occupying effect. The signal in the T2 weighted imaging was high, and in some cases inhomogeneous. In the case of a hematoma, the formation was measured according to the ellipsoid formula 0.52 × height × width × depth.

### Data analysis

The diagnosis of a rectoprostatic hematoma was correlated to the needle path, to the finding of malignancy in the tissue samples obtained, to the patient position in biopsy, to the number of cores taken, to the patient´s age, to the prostate volume, and PSA density using a multivariable logistic regression model in RStudio.

Values are given as mean ± standard deviation (SD) or as median with the interquartile range (IQR), as appropriate. A p-value of 0.05 or less was deemed statistically significant.

### Ethics approval and consent to participate

Written informed consent was obtained from all subjects (patients) in this study. Institutional Review Board approval was obtained by the ethics committee of the Witten/Herdecke University, Germany.

## Results

**Figure 4:**
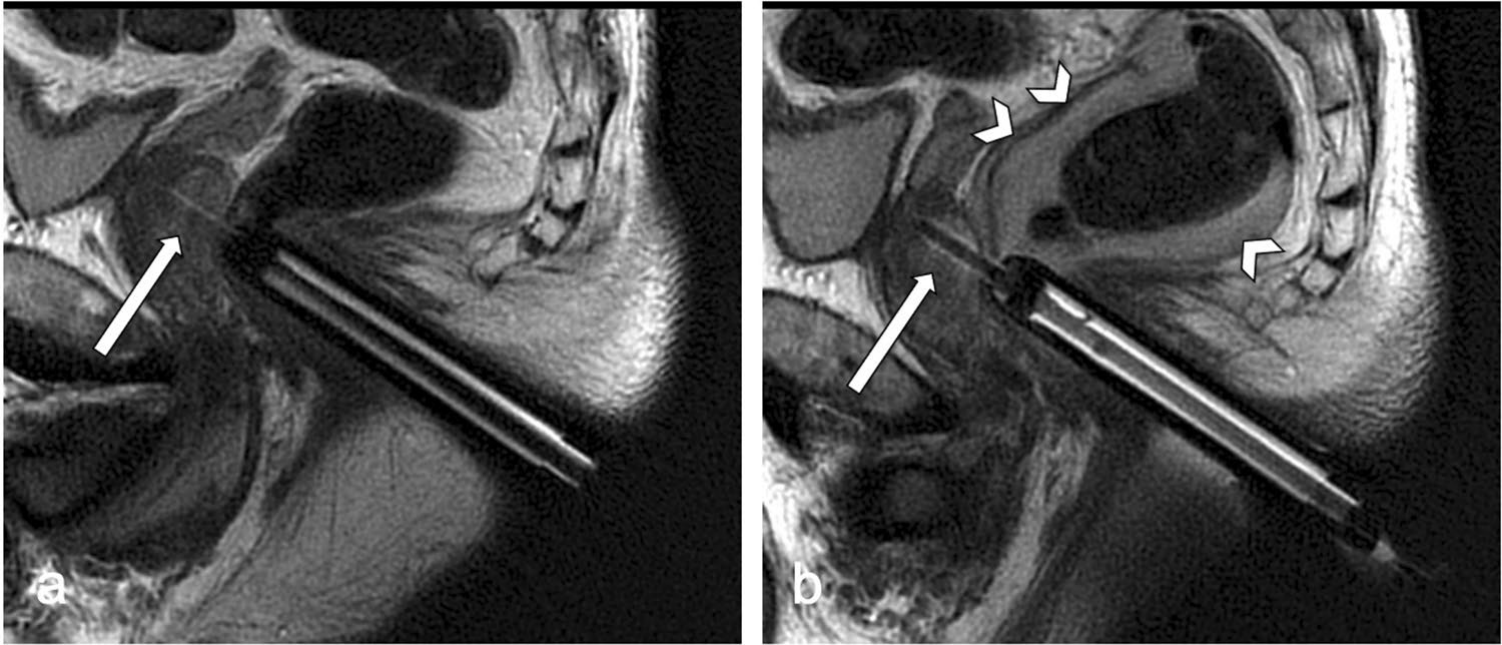
Rectal blood collection (arrowheads); sagittal T2 weighted fast imaging in a 67 years old patient with continued antiplatelet therapy; (**a**) first intraprostatic needle documentation (arrow); (**b**) needle documentation at the end of the intervention.

Evaluation could be performed in all patients (mean age 66 ± 8 years, range 39–87 years). Bleeding complications in the image documentation were detected in 41 (14.5%) of 283 patients. Rectoprostatic hematomas were found in 24 (8.5%) patients (Figs. 2, 3), a further 16 (5.7%) patients showed blood collections in the rectum (Fig. 4), one patient showed bleeding in the urinary bladder. The volume of rectoprostatic hematomas was determined with a median of 7.5 ml (range 2–40 ml, IQR 11.25).

In the patient group with rectoprostatic hematoma, 15 of 24 patients did not have any anticoagulation or antiplatelet therapy, 5 patients had continued antiplatelet therapy, one patient had an interruption of anticoagulation, in three patients there was no information available.

**Figure 5:**
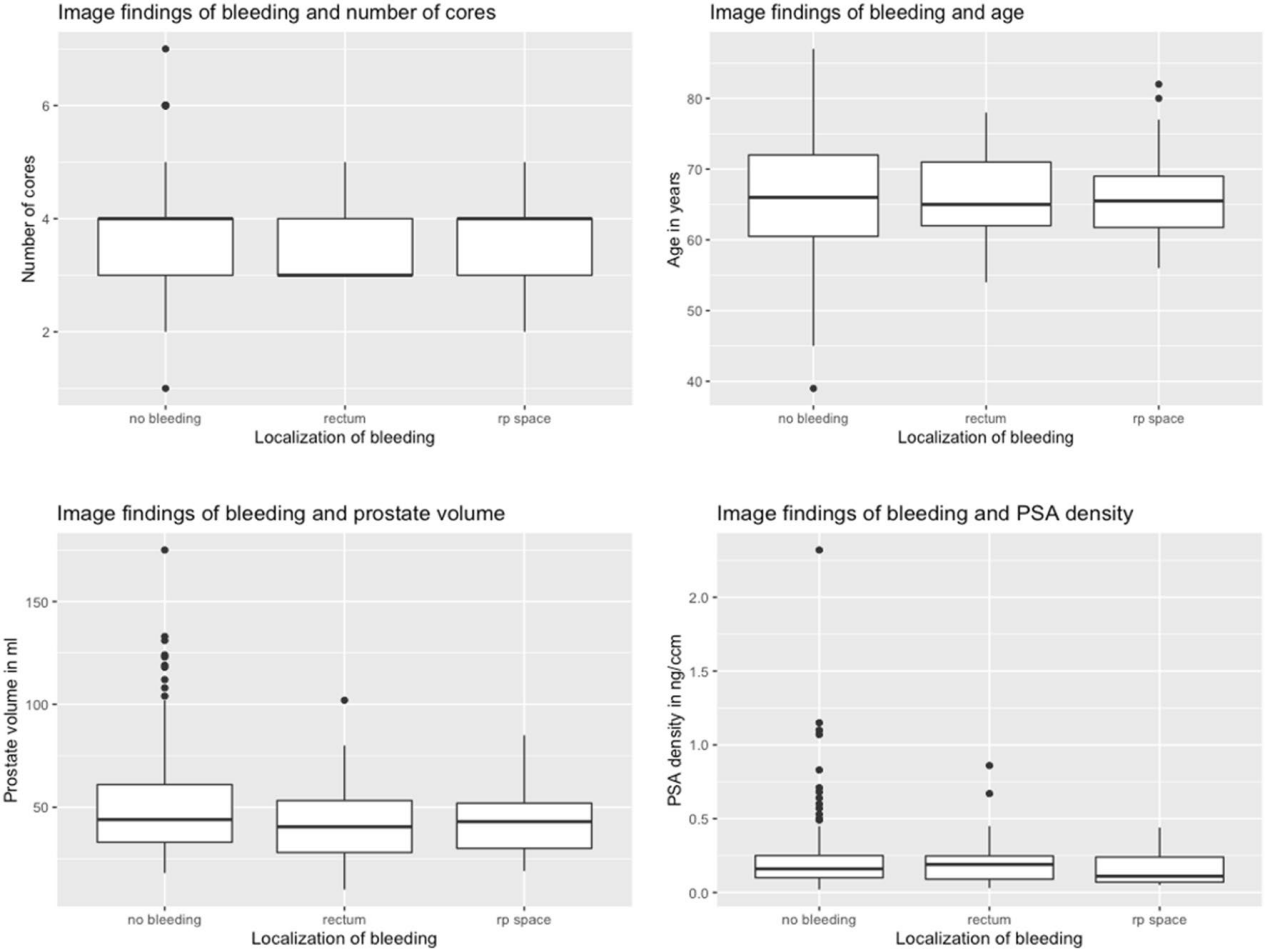
Correlation of bleeding and patient characteristics; image findings of bleeding and number of cores, age, prostate volume and PSA density, *rp space* rectoprostatic space.

Figure 5 shows the correlation between different image findings of bleeding and the number of cores, the age, the prostate volume, and PSA density. The interquartile ranges are reasonably similar for the number of cores, the age, the prostate volume, and the PSA density in different groups without bleeding, with rectal bleeding, and with rectoprostatic hematoma.

Table 2 shows the distribution of the targets to the prostate sectors for all patients and the selective distribution for patients with rectoprostatic hematoma. The posterolateral sector of the peripheral zone (PZpl) is the most lateral located sector. The needle path to targets in the PZpl was assigned to a lateral path in contrast to a medial needle path for the remaining sectors. The anterior sector of the peripheral zone (PZa) has lateral parts as well. In patients with rectoprostatic hematoma, there were no targets in the PZa.

**Table 2:** Distribution of in-bore biopsy targets to the prostate sectors.

Prostate sector	All targets n (%)	Targets in patients with rectoprostatic hematoma n (%)
Basal TZa	14 (1.4)	0 (0)
Middle TZa	49 (4.8)	2 (2.4)
Apical TZa	20 (2.0	1 (1.2)
Basal TZp	60 (5.9)	3 (3.6)
Middle TZp	105 (10.3)	11 (13.3)
Apical TZp	65 (6.4)	4 (4.8)
Basal PZa	6 (0.6)	0 (0)
Middle PZa	7 (0.7)	0 (0)
Apical PZa	7 (0.7)	0 (0)
Basal PZpm	56 (5.5)	8 (9.6)
Middle PZpm	203 (20.0)	17 (20.5)
Apical PZpm	108 (10.6)	12 (14.5)
Basal PZpl	35 (3.4)	3 (3.6)
Middle PZpl	144 (14.2)	16 (19.3)
Apical PZpl	136 (13.4)	6 (7.2)

*TZ* transition zone, *PZ* peripheral zone, *a* anterior, *pm* posteromedial, *pl* posterolateral.

Table 3 displays the characteristics of the patients and the biopsy procedure for patients with and without localized rectoprostatic hematoma.

**Table 3:** Characteristics of the patients and the biopsy procedure for patients with and without localized rectoprostatic hematoma.

Rectoprostatic hematoma	n	Lateral needle path included n (%)	Lateral targets per patient mean	Cancer yes/no	Position prone/supine	Number of cores median	Age median	Prostate volume median	PSA density ng/ccm median
Yes	24	14 (58)	1.79	16/8	7/17	4	65.5	43	0.11
No	259	162 (63)	1.75	157/102	52/207	4	66	43	0.16

The multivariable logistic regression model did not show any correlation between the presence of a rectoprostatic hematoma and the needle path, malignant findings, patient position in biopsy, number of cores, age, prostate volume nor PSA density (p > 0.05).

Three patients showed prolonged but self-limiting rectal bleeding from hemorrhoids and/or rectal polyps without periprostatic hematoma. Two of these patients had continued antiplatelet therapy. The third patient stopped antiplatelet therapy 5 days before the biopsy. Two patients developed fever after the biopsy, one of them with preexisting prostatitis. Both patients had rectal blood collection but no periprostatic hematoma. There was no further clinically relevant complication in any patient.

## Discussion

Signs of bleeding in the image documentation after prostate biopsy were detectable in 41/283 (14.5%) of all patients.

MR imaging revealed intra-rectal blood collections in 16 (5.7%) patients. Rectal bleeding is considered a less significant, self-limiting complication in TRUS biopsy in about 1.3–45% of the cases with a duration of 3 to 7 days. The definition of rectal bleeding is inconsistent. The term is used for only minimal blood admixtures in the feces but also for significant bleeding that is recognized during or after the biopsy procedure^15^. Continued use of acetylsalicylic acid may lead to prolonged duration of hematuria and rectal bleeding with varying effects on the incidence of predominantly minor hemorrhagic complications^8,18,19^. However, life-threatening situations can occur. Continuation of antiplatelet therapy has to be discussed on an individual basis^20^.

The rate of rectal bleeding may be influenced by the number of cores obtained^16^. Wegelin et al. did not find a significant difference in rectal bleeding in patients after targeted in-bore MR-guided biopsy with a reduced number of cores compared to transperineal TRUS-guided fusion biopsy and cognitive TRUS biopsy. Simultaneously, the occurrence of hematuria and hematospermia was significantly lower in targeted in-bore MR-guided biopsy compared to the fusion techniques^8^.

In another study, a lower incidence of rectal bleeding in MR-targeted biopsy compared to standard TRUS biopsy is assigned to a lower number of cores obtained in the targeted biopsy strategy, but this effect is also influenced by a lower number of biopsies, as patients with inconspicuous MR imaging did not undergo biopsy^6^.

The application of periprostatic local anesthetic does not seem to have any influence on the prevalence of rectal bleeding^19^.

The presence of rectal blood collections in the documentation may be suitable to predict the amount of visible blood in the feces. The correlation between rectal blood collections and the duration of rectal bleeding should be further evaluated.

Localized hematomas were found in 24 (8.5%) patients in the rectoprostatic space. These hematomas can be overlooked compared to rectal bleeding or hematuria. Antiplatelet therapy was continued in 5/24 patients, though this has not been evaluated systematically in this study due to the retrospective character.

So far, periprostatic hematoma has only been evaluated after manual and robotic guided transperineal biopsy under general anesthesia. Asymptomatic rectoprostatic hematomas were found in 16% of transperineal MR-manual-guided biopsy and 30% of transperineal MR-guided robotic biopsy^13^. This is higher than 8.5% in our study population without general anesthesia, and this is associated with a higher number of cores compared to the procedure in our patients. The hematomas did not require further intervention.

In contrast to some TRUS biopsy procedures, we did not use a periprostatic nerve block in in-bore MR-guided biopsy. To date, there are no data available in the literature on the rate of rectoprostatic hematomas in TRUS-guided biopsy. The influence of the number of cores remains unclear.

The neurovascular bundle contains the prostatic artery, veins, and nerves from the prostatic plexus. The neurovascular bundle enters the prostate bilaterally at the rectoprostatic angle. The prostatic venous plexus is located laterally and anteriorly to the prostate. The inclusion of lateral prostate sectors using a lateral needle path did not show an increased risk for rectoprostatic hematoma in our study. The location of hematoma in the rectoprostatic space seems to be predominantly based on communicating veins from the rectoprostatic space and the rectum with the prostatic plexus^21^. The proportion of rectoprostatic hematoma was slightly higher for patients in the prone position (3 Tesla) compared to patients in the supine position (1.5 Tesla). For the small number of patients in the prone position in our study, this difference did not reach significance. With a view to the vessel anatomy, it can be assumed that the patient´s position may influence venous congestion. Finally, the influence of the magnetic field strength cannot be excluded.

In the literature there are further complications described in prostate biopsy. Hematuria and hematospermia are reported with a rate of up to more than 90% of patients after prostate biopsy. There are equivocal findings regarding the incidence of hematospermia in correlation to the number of cores taken^15^.

Temporary erectile dysfunction is described after prostate biopsy in 11–26% using different approaches and completely recovers after 1–3 months. The etiology needs to be further clarified^6,7,10,16,22^.

Intensity and duration of pain were significantly lower in patients undergoing in-bore MR-guided biopsy compared to TRUS biopsy^6,11^. Pain can be localized in the rectum or the perineum according to the utilized approach. The reduced number of cores seems to be the reason for less pain in in-bore biopsy compared to TRUS biopsy. To date, the presence of a hematoma has not been evaluated in this context.

Urinary retention is not a common complication after a transrectal biopsy. Urinary retention is predominantly described in transperineal mapping biopsies with a high number of cores^10^. In our study population, no patient was showing urinary retention.

Infection may have a prominent clinical role. The rate of infection is reported from 0.1 to 7%, and the rate of sepsis ranges from 0.3 to 4.2%. The contributing factors are predominantly the approach for biopsy, either transrectal or transperineal and the number of cores^2,8,16^. We found a low rate of infection with 2/283 patients (0.7%). There seems no plausible correlation between infection and bleeding or hematoma.

Overall, transrectal in-bore MR-targeted biopsy demonstrates a favorable rate of minor complications compared to fusion biopsies and standard TRUS biopsies^6,8,11^. To date, there are no data about the meaning of rectal blood collections in the image documentation for the postprocedural course nor data about the possible role of periprostatic hematoma for pain, erectile dysfunction, and urinary retention. In the awareness that rectoprostatic hematoma may occur after prostate biopsy, further studies can focus on such correlations.

There are some limitations. Due to the retrospective character of this study, we did not analyze visible bleeding from the rectum or preceding anticoagulation systematically. Pain scores and erectile dysfunction were not evaluated.

No follow-up scans in MR imaging are available. The time course of the hematoma remains unclear.

The existing needle control images were searched for hematomas, there was no specific image control with a focus on a hematoma. If a hematoma had been present in an area outside the imaging focus, an undersampling of hematomas would have been possible. Nevertheless, needle control was systematically performed in two planes for each core obtained.

During this long period of study time, the underlying PI-RADS score was developed and modified. Nevertheless, all patients were retrospectively reassessed according to PI-RADS version 2.1.

## Conclusions

Bleeding complications are scarce after an in-bore MR-guided prostate biopsy.

MR imaging provides benefits not only for lesion detection in prostate biopsy but also for the control of bleeding complications, which can be overlooked in standard TRUS biopsy. Rectoprostatic hematomas and rectal blood collections can be easily detected and documented by MR imaging.

In this study was no correlation between bleeding complications and patient demographics, clinical characteristics, nor the biopsy procedure. The rectoprostatic hematomas are predominantly clinically silent, their relevance concerning pain, erectile dysfunction, and urinary retention remains to be investigated.

## Data Availability

The datasets generated during and/or analysed during the current study are not publicly available due, as they contain patient data, but are available from the corresponding author on reasonable request.

## Abbreviations

PSA: Prostate-specific antigen
rp space: Rectoprostatic space
TRUS biopsy: Transrectal ultrasound-guided biopsy
